# Broad distribution spectrum from Gaussian to power law appears in stochastic variations in RNA-seq data

**DOI:** 10.1038/s41598-018-26735-4

**Published:** 2018-05-29

**Authors:** Akinori Awazu, Takahiro Tanabe, Mari Kamitani, Ayumi Tezuka, Atsushi J. Nagano

**Affiliations:** 1 Department of Mathematical and Life Sciences, Hiroshima University, Kagamiyama 1-3-1, Higashi-Hiroshima, Hiroshima, 739-8526 Japan; 2 Research Institute for Food and Agriculture, Ryukoku University, Yokotani 1-5, Seta Ohe-cho, Otsu, Shiga, 520-2194 Japan; 3 Faculty of Agriculture, Ryukoku University, Yokatani 1-5, Seta, Ohe-cho, Otsu-shi, Shiga, 520–2194 Japan; 4 Research Center for Mathematics on Chromatin Live Dynamics, Hiroshima University, Kagamiyama 1-3-1, Higashi-Hiroshima, Hiroshima, 739-8526 Japan

## Abstract

Gene expression levels exhibit stochastic variations among genetically identical organisms under the same environmental conditions. In many recent transcriptome analyses based on RNA sequencing (RNA-seq), variations in gene expression levels among replicates were assumed to follow a negative binomial distribution, although the physiological basis of this assumption remains unclear. In this study, RNA-seq data were obtained from *Arabidopsis thaliana* under eight conditions (21–27 replicates), and the characteristics of gene-dependent empirical probability density function (ePDF) profiles of gene expression levels were analyzed. For *A. thaliana* and *Saccharomyces cerevisiae*, various types of ePDF of gene expression levels were obtained that were classified as Gaussian, power law-like containing a long tail, or intermediate. These ePDF profiles were well fitted with a Gauss-power mixing distribution function derived from a simple model of a stochastic transcriptional network containing a feedback loop. The fitting function suggested that gene expression levels with long-tailed ePDFs would be strongly influenced by feedback regulation. Furthermore, the features of gene expression levels are correlated with their functions, with the levels of essential genes tending to follow a Gaussian-like ePDF while those of genes encoding nucleic acid-binding proteins and transcription factors exhibit long-tailed ePDF.

## Introduction

Stochastic variations in gene expression—known as gene expression noise or phenotype fluctuation—have been observed among individuals in a genetically identical population under the same environmental conditions^1–11^. Such variations are thought to be important for maintaining the pluripotency of embryonic stem cells, cell fate decisions, and cellular differentiation in multicellular organisms^12–15^. In rice, genes related to stress responses exhibited larger variations than those involved in other processes^16^. Furthermore, recent studies in *Escherichia coli*, the budding yeast *Saccharomyces cerevisiae*, and *Arabidopsis thaliana* have reported that the magnitude of gene expression noise is positively correlated with plasticity—i.e., the variation in expression levels due to mutation or environmental change^17–27^.

Recent gene expression analyses with sufficiently large replicates have shown that in organisms as diverse as *E. coli* and mammals, fluctuations in protein expression level for a given gene follow a log-normal distribution^2,6,7,17^. The closely related Frechet distribution was also proposed to describe variations in gene expression levels in *E. coli* and *S. cerevisiae*^28^. On the other hand, mathematical modeling of protein expression in *E. coli* suggested that such variations were more closely approximated by a gamma distribution, which is often considered as log-normal^8,29^.

In many recent high-throughput RNA sequencing (RNA-seq) studies^30,31^, variations in gene expression (transcription) levels among replicates were assumed to follow a log-normal distribution^32,33^ or a negative binomial (NB) distribution^34–36^. An analysis of RNA-seq data from a two-condition, 48-replicate experiment using *S. cerevisiae* revealed that variations in expression levels for each gene conformed to both log-normal and NB distributions^36^. Beta-binomial and Benford distributions have been proposed for fitting gene expression data obtained by RNA-seq^37,38^. However, the physiological basis of these distributions and the significance of associated parameters remain unclear. Furthermore, it is not known whether such model distributions are applicable to any genes in any organism, especially multicellular organisms.

Gene expression noise in plants has been investigated in rice and *Arabidopsis*^16,27,39^. However, recent studies were based on transcriptome data from experiments with few replicates^40–45^, which limited the inferences that could be made regarding the distribution characteristics of gene expression levels. In the present study, we analyzed RNA-seq data for *A. thaliana* under eight conditions (21–27 replicates) to observe the empirical probability distribution profiles of gene expression levels among individuals in a homogeneous population. We fitted the distribution profiles with a novel function that we termed the Gauss-power (G-P) mixing distribution function, which was derived from a simple stochastic transcriptional network model containing a feedback loop. Moreover, the features of each probability distribution function in gene expression level are expected to be correlated with the strength of feedback regulation for each gene, its average expression level, and its function.

## Results

### Analysis of Arabidopsis RNA-seq data

RNA-seq data from 7- and 22-day-old *Arabidopsis* shoots cultured under a 12:12-h light/dark cycle were obtained 1, 7, 13, and 19 h after the lights were turned on. There were 21 to 27 replicates for each condition. In total, 189 individual plants were analyzed by RNA-seq. We obtained 8.4 million reads on average; one sample with fewer than 1 million reads mapped to genes was omitted from subsequent analyses. The expression level was quantified according to a previously described pipeline^46^ (Table S1). For each condition, we examined the distribution profiles of expression levels of ~10,000 genes (Table 1) whose expression levels could be regarded as stationary (see Materials and Methods).

**Table 1:** Number of replicates in the RNA-seq experiment and number of analyzed genes for each condition of *Arabidopsis*.

Condition	Age (days)	Time after light (h)	Replicate	No. of analyzed genes
7-1	7	1	21	10,499
7-7	7	7	21	9287
7–13	7	13	25	10,760
7–19	7	19	24	10,735
22-1	22	1	22	9619
22-7	22	7	24	12,109
22-13	22	13	24	12,810
22-19	22	19	27	11,338

### Cluster analysis of empirical cumulative distribution function (eCDF) profiles of genes

**Figure 1:**
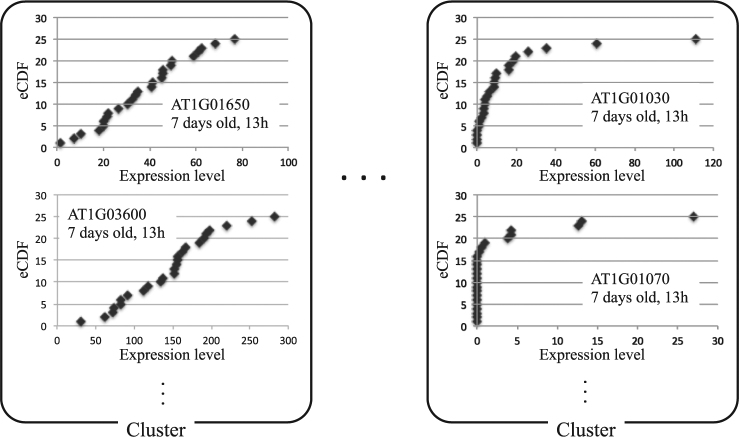
eCDF profiles of genes in respective clusters. eCDF profiles of indicated genes under specific conditions. As examples, results obtained from RNA-seq data at 13 h for 7-day-old *Arabidopsis* are shown. Similar RED profiles were grouped by cluster analysis.

eCDF profiles were obtained for each gene under each condition (harvest time and age of plant) by arranging its expression levels in ascending order (Fig. 1). For most genes, the eCDFs showed typical profiles but were very noisy. We then performed a cluster analysis to estimate an ideal curve of eCDF profiles from which noise has been removed for each gene (see Materials and Methods).

**Figure 2:**
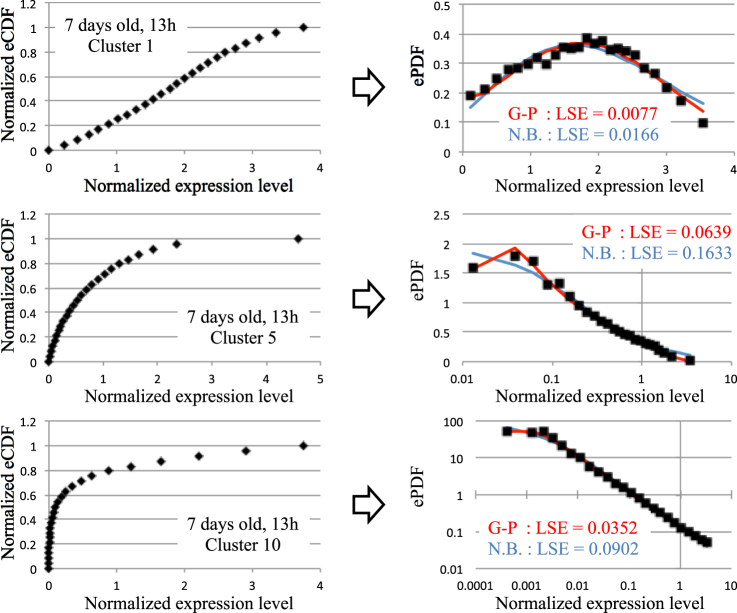
Examples of eCDF and ePDF for indicated clusters. Representative profiles of eCDF (left) and ePDF (right) as a function of normalized expression levels for three clusters. Red and blue represent curves fitted with the G-P and NB distribution functions, respectively. Least square error was estimated for the fitting curves.

For each condition, 12–15 clusters were obtained from normalized eCDF profiles (Tables S2 and S3), representing the relationship between standardized expression levels for mean = 0 and standard deviation = 1 and normalized eCDF from 0 to 1. The average values of standardized expression levels of genes belonging to the same cluster were expected to reflect the essential features of their eCDF profiles. Thus, these average values were considered in order to estimate and analyze eCDF profiles of each gene. Since gene expression levels are non-negative, we analyzed normalized eCDFs that were shifted such that the minimum value on the horizontal axis was assumed to be 0 (the value on the horizontal axis represents normalized expression level) (upper and lower left in Fig. 2).

### Inferences on probability density distribution profiles of gene expression levels

The derivative of eCDF yielded the profiles of empirical probability density function of normalized expression levels (ePDF) for each cluster (Figs 2 and S1–S8). The derivative of this function was estimated by differential approximation (see Supplementary Information S1). ePDF profiles obtained from the clusters showed variable shape, including Gaussian and power law-like distributions.

### G-P mixing distribution function

The ePDF profiles of gene expression levels were systematically classified based on the following mathematical model. A novel probability density function, which we refer to as Gauss-power mixing function (G-P function), is described by equation 1.

1 $$P(x)=A\frac{K+x}{fx}{x}^{\frac{2gK-{K}^{2}}{{f}^{2}}}{e}^{-\frac{1}{{f}^{2}}[\frac{g{K}^{2}}{x}+(2K-g)x+\frac{{x}^{2}}{2}]}\,$$

This equation is a fitting function of the probability density function of expression level *x* of the gene of interest *X*; the parameter *A* is a normalized coefficient; and *f*, *g*, and *K* are constants whose physiological significance is described below.

**Figure 3:**
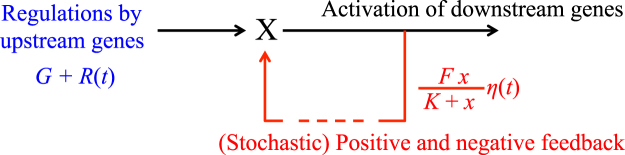
Illustration of a gene network model that fits a G-P function. Gene *X* is regulated by upstream genes and by stochastic feedback.

In general, the expression levels of genes are increased by activation and decreased by inhibition of upstream genes, and influence the expression levels of their downstream targets in a gene regulatory network. They are also positively or negatively regulated by the expression levels of their downstream targets either directly or indirectly because the gene regulatory network includes many positive and negative feedback loops (Fig. 3). Thus, the expression level of each gene may regulate itself through such feedback loops. Furthermore, gene expression levels always exhibit and are influenced by stochastic noise to a degree that is correlated with the levels themselves.

Thus, a simplified model of the temporal change in the expression level *x* of gene *X* influenced by upstream genes and feedback regulation (Fig. 3) is given by equation 2:

2 $$\frac{dx}{dt}=G+R(t)+\frac{Fx}{K+x}\eta (t)-Cx$$

where *R*(t) and $\eta$(*t*) are assumed to be Gaussian white noise with $\langle R(t)\rangle =\langle \eta (t)\rangle =0$, $\langle R(t)R(t^{\prime} )\rangle =2D\delta (t-t^{\prime} )$, and $\eta (t)\eta (t^{\prime} )=2\delta (t-t^{\prime} )$; the parameters *G*, *K*, *F*, and *C* are ~ [average activation rate of *X* by upstream genes], ~ [average expression level of *X* required to induce maximum expression of downstream genes], ~ [magnitude of feedback effects], and [degradation rate of *X*], respectively. If *D/C*→0 is assumed, P(x) gives the steady-state probability distribution of *x* where *g* = *G*/*C* and *f* = *F*/*C* (see Supplementary Information S2).

It is worth noting that the parameter *K* can be eliminated from Eq. (2) by appropriate variable conversion that decreases the number of parameters in the model. From this model, a G-P function with two parameters only can be derived. However, in such a two-parameter-model, a specific scale of *x* is given in the process of elimination of K. Thus, generally Eq. (1) appears to be appropriate for the fitting and analysis of ePDF profiles. Moreover, *K* and *F* (or *F/K*) characterize the feedback effect, similar to the Michaelis constant and maximum reaction velocity, respectively, of various biochemical reactions. We, therefore, included these parameters in the present argument.

### Fitting of ePDF profiles

ePDF profiles of each cluster under each condition were fitted with the G-P function and (generalized) NB function according to equation 3:

3 $$N(x)=B\frac{{\rm{\Gamma }}(sx+k+1)}{{\rm{\Gamma }}(k){\rm{\Gamma }}(sx+1)}{Q}^{k}{(1-Q)}^{sx}$$

where Γ(*r*) is the gamma function; *s*, *k*, and *Q* are fitting parameters; and *B* is a normalized coefficient. Note that the parameter *s*—which is usually equal to 1—contributes to the generalization for various scales of *x*.

The characteristics of ePDF profiles for some clusters can be extracted from plots with a linear scale axis; however, it is more difficult to extract those of profiles with much larger maximum values and that exhibit power law-like profiles, for which log-log plots seem more suitable when the maximum ePDF value is greater than 3. In order to extract their detailed characteristics, ePDF profiles were fitted using a typical least squares method for maximum ePDF values <3; ePDF fitting parameters were chosen so as to minimize the sum of squared errors between log[ePDF] and log[fitting functions] when the maximum ePDF value was >3.

The results of fitting by the G-P and NB functions suggest that the G-P function has a least square error that tends to be smaller than that of the NB function for ePDF profiles of most clusters (Fig. 2 and Table S3), whereas the G-P function has a smaller LSE than the NB function for ~82% of ePDF profiles of clusters (86/105 clusters). It should be noted that both G-P and NB functions include the same number of parameters. Thus, the former provides a function that better fits ePDF profiles than the latter. Therefore, in subsequent analyses the ePDF profiles were classified according to a G-P function.

### Classification of ePDF profiles

**Figure 4:**
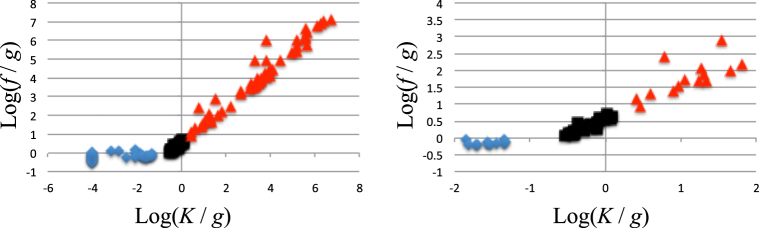
Relationships among fitting parameters of the G-P function from each ePDF profile of each cluster. Scatterplot of *f*/*g* and *K*/*g* of the G-P function that fits each ePDF profile of each cluster. Overall image (right) and local image near the origin (left). (*f*/*g*, *K*/*g*) clearly distinguish three groups—i.e., log(*K*/*g*), K = 0 or log(*K*/*g*) <−1.3 (blue), −0.6 <log(*K*/*g*) <0.1 (black), and log(*K*/*g*) >0.4 (red). For convenience, when K = 0, log (*K*/*g*) = log (10^−4^/*g*) is plotted.

When ePDF profiles of each cluster were fitted with the G-P function, they were classified by three clearly divided groups with different log(*K*/*g*), K = 0 or log(*K*/*g*) <−1.3, −0.6 <log(*K*/*g*) <0.1, and log(*K*/*g*) >0.4 (Fig. 4 and Table S3). Here, log(*f*/*g*) showed a strong positive correlation with log(*K*/*g*) in second and third groups of log(*K*/*g*) (Fig. 4). From Eq. (2), the power-law distribution of *x* was obtained over a wide range of log *x* in the case of a much larger *K*/*g* (and *f*/*g*), while a Gaussian distribution was obtained in the case of a much smaller *K* (see Supplementary Information S2). Thus, a G-P function with *K* ≫ *g* was closer to a power law distribution. Based on these facts, ePDF profiles could be classified as one of three types: Gaussian (*K* ≪ *g*), power law-like (*K* ≫ *g*), or intermediate (*K* ≈ *g*) (Table S3). When the influence of feedback effects is large relative to other mechanisms regulating gene expression, gene expression levels exhibit a long-tailed power law-like distribution.

**Figure 5:**
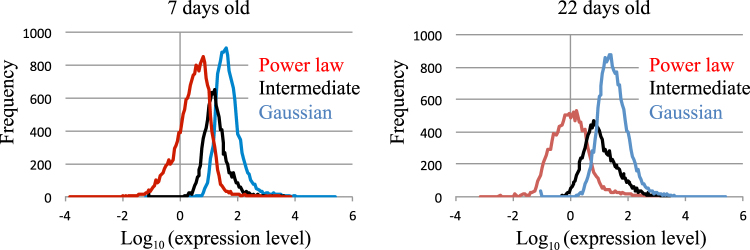
Frequency distributions of average gene expression levels of gene groups exhibiting distinct ePDF profiles. Frequency distributions of average log gene expression levels in cases of Gaussian (blue), intermediate (black), and power law-like (red) distributions in 7-day-old (left) and 22-day-old (right) *Arabidopsis*. Differences in average log gene expression levels between Gaussian and intermediate, and between mixed and power law-like distributions were significant (P < 0.01, t test) at both plant ages.

Even for the same gene, ePDF profiles varied depending on plant age and harvest time (Table S2). In particular, genes exhibiting an intermediate ePDF profile at one time point tended to exhibit other profiles at other time points (Table S2). The ratio of occurrence of Gaussian, intermediate, and power law-like distributions at four time points in younger plants (7 days old) was ~30:26:44, while that of older plants (22 days old) was ~45:29:26. High average expression levels were more frequently associated with a Gaussian as compared to a power law-like distribution; average expression levels and peak value of the frequency distribution were higher for the former than for the latter (Fig. 5). However, it was difficult to clearly classify each gene based solely on the mean expression level, since ePDF profiles of genes with moderate mean expression levels exhibit Gaussian, intermediate, or power-law like distribution.

Gene function was also correlated with ePDF profiles (Tables 2 and S4). For example, more than half of so-called essential genes^46^ showed a Gaussian distribution at four time points in young and old plants. Furthermore, genes encoding important intracellular components and organelles and those associated with electron transport in metabolic pathways tended to show Gaussian distributions. On the other hand, genes encoding transcription factors and nucleic acid-binding proteins mostly exhibited power law-like and intermediate distributions.

**Table 2:** Relationships between gene groups classified according to function and ratio of occurrence of ePDF profiles.

	7 days old		
	Power	Intermediate	Gaussian
All genes	0.435357671	0.260434582	0.304207747
Essential genes	0.208732694	0.248136315	0.54313099
GO annotated genes	0.322610702	0.276288934	0.401100364
(GO slim)			
Mitochondria	0.464503043	0.249492901	0.286004057
Extracellular	0.45	0.246206897	0.303793103
Plastid	0.149706795	0.245946878	0.604346326
Cytosol	0.205555556	0.283597884	0.510846561
Ribosome	0.216624685	0.258186398	0.525188917
Transcription factor activity	0.484303056	0.239849309	0.275847635
Nucleic acid binding	0.458526875	0.262773723	0.278699403
Other molecular functions	0.448415374	0.224544842	0.327039784
Structural molecule activity	0.204889406	0.263096624	0.53201397
Electron transport or energy pathways	0.154351396	0.238095238	0.607553366

## Discussion

More than 20 replicates of *A. thaliana* gene expression data at four harvest times of 7- and 22-day-old shoots were obtained and the ePDF profiles of each gene were analyzed. Most profiles could be fitted with a G-P function. Each gene could be classified by parameters of the G-P function fitting its ePDF profile of expression levels. There were three typical ePDF profiles—namely, Gaussian, power law-like, and intermediate.

The G-P function suggested that the various types of ePDF profile were highly correlated with network topology, particularly a feedback loop regulating gene expression; for instance, gene groups showing a power law-like distribution were predicted to be significantly influenced by a feedback mechanism, while this was rare for those exhibiting a Gaussian distribution.

In the present argument, Eq. (1) was obtained under the assumption that *D*/*C* is considerably smaller than any of the other values for the sake of simplicity. Equation (1) can play an important role in unambiguously classifying genes based on their ePDF profiles. On the other hand, the Gaussian distribution can be derived from Eq. (2) in cases not only such as (i) *K* ~ 0 and *F*/*C* > 0 (*D*/*C* ~ 0) as shown above, but also those such as (ii) *F* = 0 and *D*/*C* > 0. For some genes, for example those without any downstream, Eq. (2) with F = 0 and D/C > 0 may provide a more suitable model for the fluctuation of transcription levels.

There are other possible interpretations and mechanisms for a G-P function. In addition, Eq. (2) should be derived based on the general features of gene regulatory networks to confirm its validity as an effective model of gene regulatory dynamics. Relevant studies are now underway.

The ePDF profiles of genes were correlated with their average expression levels and functions; gene groups classified as being essential for survival tended to exhibit Gaussian distributions, whereas those encoding transcription factors and nucleic acid-binding proteins mostly followed a non-Gaussian (i.e., power law-like or intermediate) distribution. Furthermore, the expression levels of many genes classified as “unknown” exhibited power law-like distributions (Table S4), suggesting that their expression is predominantly modulated by feedback loops.

ePDF profiles of gene expression levels were inferred from publicly available RNA-seq data derived from 48-replicate experiments of *S. cerevisiae*^36^ in the same manner as in the present study. The G-P as well as the NB function fit the ePDF profiles of *S. cerevisiae* (Fig. S9). However, long-tailed power law-like ePDF profiles were not observed unlike for *Arabidopsis* genes for reasons that are unclear. There are many differences between *Arabidopsis* and *S. cerevisiae*: the former is a multicellular organism that undergoes differentiation, in which genes different genes are required by different cell types; it also exhibits circadian rhythm, with gene expression profiles showing variations over time; and finally, as a plant it has complex metabolic and gene regulatory networks that allow it to adapt to environmental stresses. Thus, *Arabidopsis* is expected to have more complex gene regulatory networks that include extensive feedback regulation than yeast; thus, some *Arabidopsis* genes can exhibit power-law like ePDF, unlike those of yeast.

Even when the analysis was performed using 24-replicate data randomly selected from the 48-replicate dataset, the results were qualitatively similar to those described above, except that the number of clusters differed (Fig. S10). Although the number of replicates in the present study was smaller than that used in the earlier report, our results reflect the essential properties of the ePDF profiles of *Arabidopsis* genes and are expected to apply to a larger number of replicates.

The present study showed that the expression levels of some *Arabidopsis* genes often exhibit power-law like distribution profiles that have not been reported in earlier RNA-seq analyses of various organisms. To obtain a long-tailed power law-like ePDF, a sufficient amount of rare data exhibiting a much higher expression levels than other genes must be observed. Such rare data have mostly been neglected as outliers in recent RNA-seq analyses with two or three replicates. On the other hand, the present RNA-seq analysis with more than 20 replications should provide a sufficient amount of such rare data that constitute the long-tail portion of ePDF profiles.

As shown in Fig. 1, the eCDF of transcription levels of each gene did not follow a smooth curve, but had a staircase-like slope. Accordingly, the obtained ePDF profile was too jagged for fitting and analysis. In the present study, the expression levels of different genes in the same cluster were mixed in order to simultaneously estimate and analyze ePDF profiles of each gene. However, it is possible that the present analysis contains some statistical bias. In order to test the validity of the present method and estimate such bias, it is necessary to analyze more data on the expression levels under identical conditions that can estimate the ePDF profile of each gene. Experiments involving larger replicates and additional statistical methods to analyze the present data are being developed and will be reported in the future.

In the present study, *Arabidopsis* genes were clearly separated into three classes. However, the underlying mechanistic and physiological reasons remain unclear; this issue should be analyzed in future.

This study mainly focused on the steady-state probability distributions of gene expression levels. However, many *Arabidopsis* genes are regulated by circadian rhythm. Future studies must therefore address the extent to which the present model can be generalized to dynamic situations. Furthermore, eq. (2) represents the expression of genes following a power law-like distribution, which is considered to exhibit intermittent temporal changes. This fact suggests a novel transcriptional burst mechanism^8,9,47–50^ for genes based on feedback regulation. Such dynamic features of gene expression warrant more detailed examination.

## Materials and Methods

### Plant growth conditions and RNA-seq

Seeds of *A. thaliana* (accession Col-0) were sown on Murashige and Skoog medium with 0.5% gellan gum. After incubation for 2 days at 4 °C in dark, the seeds were cultivated at 22 °C on a 12:12-h light/dark cycle. The whole aerial part of plants 7 or 22 days after germination was collected 1, 7, 13, and 19 h after the start of light period and immediately frozen in liquid nitrogen and stored on −20 °C until RNA extraction. Each individual plant was used as a sample for RNA-seq. Total RNA was extracted with the Maxwell 16 LEV Plant RNA kit (Promega, Madison, WI, USA). RNA-seq library preparation was performed as previously described^51^; seven lanes of single-end 50-bp sequencing of the library were analyzed using the Hiseq. 2000 and HiSeq. 2500 systems (Illumina, San Diego, CA, USA). Sequences were pre-processed, mapped, and quantified according to a previously described pipeline^46^. Fastq files were deposited into the DNA Data Bank of Japan Sequence Read Archive as accession no. DRA005887. Quantified expression data are available as Supplementary Information (Table S1).

### Analysis of gene expression level variation bias

Owing to technical limitations, there was a time lag of several to 10 min during the harvesting of *Arabidopsis* leaf samples, potentially introducing a bias in the expression levels of some genes with respect to harvest time. In order to evaluate the variation bias in gene expression levels under each condition, we calculated the average gene expression levels from half of the samples harvested at early time points and half harvested at late time points. Gene expression level was regarded as stationary (unbiased) if the P value in the t test was >0.2.

### Cluster analysis

k-Means cluster analysis using R software (http://www.r-project.org) was performed for normalized eCDF profiles using the Eucidian metric. The number of clusters was selected so as to minimize the Bayesian information criterion.

### Data sources for gene classification

To classify each gene, the Gene Ontology Slim classification list was obtained from TAIR (http://www.arabidopsis.org). Data on essential genes were obtained from the SeedGenes Project (http://www.seedgenes.org/GeneList)^52^.

## Electronic supplementary material

Supplementary information

Table S1

Table S2

Table S3

Table S4
