# Supplementary material for: Broad distribution spectrum from Gaussian to power law appears in stochastic variations in RNA-seq data
Source: Sci Rep. 2018 May 29;8:8339. doi: 10.1038/s41598-018-26735-4 (PMC5974282; doi:10.1038/s41598-018-26735-4)
Supplement: Supplementary file 1 — Supplementary information [file 41598_2018_26735_MOESM1_ESM.pdf]

Supplementary information for:

Broad distribution spectrum from Gaussian to power law appears in stochastic variations in RNA-seq data

Authors: Akinori Awazu[1,4,*], Takahiro Tanabe[1], Mari Kamitani[2], Ayumi Tezuka[2], Atsushi J. Nagano[2,3]

Text S1-S2

S3: Legends for Supplementary Tables S1–S4

S4: Supplementary Figures S1–S10

S1 Differential approximation of empirical probability density function derivatives according to expression levels

The normalized empirical cumulative distribution function $eCDF(e_n)$ is a discrete function where e_n indicates n-th smallest value of normalized expression levels. Profiles of the empirical probability density function $ePDF(e'_n)$ at $e'_n = (e_n + e_{n+1})/2$ were estimated by differential approximation as follows:

$$ePDF(e'_n) = \frac{eCDF(e_{n+1}) - eCDF(e_n)}{e_{n+1} - e_n} \qquad (S1).$$

S2 Derivation of P(x) from the reaction network model

From the stochastic differential equation

$$\frac{dx}{dt} = G + R(t) + \frac{Fx}{K + x}\eta(t) - Cx \qquad (S2)$$

where $R(t)$ and $\eta(t)$ are assumed to be Gaussian white noise with $\langle R(t)\rangle = \langle \eta(t)\rangle = 0$, $\langle R(t)R(t')\rangle = 2D\delta(t - t')$, and $\langle \eta(t)\eta(t')\rangle = 2\delta(t - t')$, and G, K, F, and C are constant values. The Fokker-Plank equation for the probability density distribution of x, $P(x, t)$, through the Stratonovich interpretation for $D/C = 0$ is given as

$$\frac{\partial P(x,t)}{\partial t} = \frac{\partial}{\partial x}\left(-(g - x)P(x,t) - \frac{1}{2}\left(\frac{\partial}{\partial x}\frac{f^2x^2}{(K+x)^2}\right)P(x,t) + \frac{\partial}{\partial x}\left(\frac{f^2x^2}{(K+x)^2}P(x,t)\right)\right) \qquad (S3)$$

where $g = G/C$ and $f = F/C$. The steady-state probability density distribution of x, $P(x)$ followed the equation

$$-(g - x)P(x) + \frac{1}{2}\left(\frac{\partial}{\partial x}\frac{f^2x^2}{(K+x)^2}\right)P(x,t) + \frac{f^2x^2}{(K+x)^2}\frac{\partial P(x)}{\partial x} = 0 \qquad (S4).$$

As a solution for this differential equation, $P(x)$ is given as a Gauss-power mixing distribution function as Eq. (1).

Note that Eq. (S2) with $D = 0$ can be transformed at the limit $\frac{g}{K} \to 0$ to

$$\frac{d}{dt}\left(\frac{x}{K}\right) = \frac{f}{K}\frac{\frac{x}{K}}{1+\frac{x}{K}}\eta(t) - \frac{x}{K} \qquad (S5)$$

Then,

$$\frac{dx}{dt} = \left(\frac{f}{K}\eta(t) - 1\right)x \qquad (S6)$$

was obtained for $x \ll K$. In the manner described above, the steady-state probability density distribution for $x \ll K$ for K much larger than g obeys the power-law as follows:

$$P(x) \propto x^{-\left(1+\frac{K^2}{f^2}\right)} \qquad (S7).$$

On the other hand, when $x \gg K$,

$$\frac{dx}{dt} = f\eta(t) - x \qquad (S8)$$

was obtained. The steady-state probability density distribution for $x \gg K$ obeys a Gaussian distribution as

$$P(x) \propto e^{-\frac{x^2}{2f^2}} \qquad (S9).$$

The mean stochastic feedback in Eq. (S2) was assumed to be 0. This model can be extended to more general cases with non-zero mean of stochastic feedback as follows:

$$\frac{dx}{dt} = G + R(t) + \frac{H + F\eta(t)}{K + x} x - Cx \qquad (S10)$$

with $H \neq 0$. For $x \ll K$, Eq. (S10) can be transformed as follows:

$$\frac{dx}{dt} = G + R(t) + \frac{F}{K} x\eta(t) - \left(C - \frac{H}{K}\right) x \qquad (S11),$$

which can then be transformed to

$$\frac{dx}{dt} = (G + H) + R(t) + \frac{Fx}{K + x} \eta(t) - Cx \qquad (S12)$$

for $x \gg K$. This indicates an increase and decrease in H have effects similar to the increase and decrease in G/C in Eq. (S2).

S3 Supplementary Tables (legends)

Table S1. Expression levels of *Arabidopsis* genes (21–27 replicates) under each condition. Day_hour is the age of harvested plants, where data at 7_1 were obtained from 7-day-old plants 1 h after the lights were turned on. # of reads indicates total reads (mega reads) of each RNA-seq experiment (M reads). Replicate indicates the index of data from each experiment. AT…G… indicates the gene name (lower left) and each number (lower right) shows the expression level of each gene (RPKM).

Table S2. Names of gene clusters and types of ePDF profile for each condition. 7_1–22_19 correspond to conditions (Day_hour) of harvested plants. Gene name (left box), index of the cluster involving each gene (middle box), and classified type of ePDF profile (right box) under each condition of harvested plant are shown. A blank indicates that the expression level of the gene was biased under a given condition.

Table S3. Number (#) of genes; least-square errors of fitting of ePDF profiles by G-P and NB functions ([Sq. Err. G-P] and [Sq. Err. N.B]); parameters [A, g, f, and K] for G-P mixing distribution; log(f/g) and log(K/g); and type of distribution for each cluster under each condition.

Table S4. Ratios of occurrence of ePDF profiles for gene groups classified by Gene Ontology slim terms.

S4 Supplementary figures

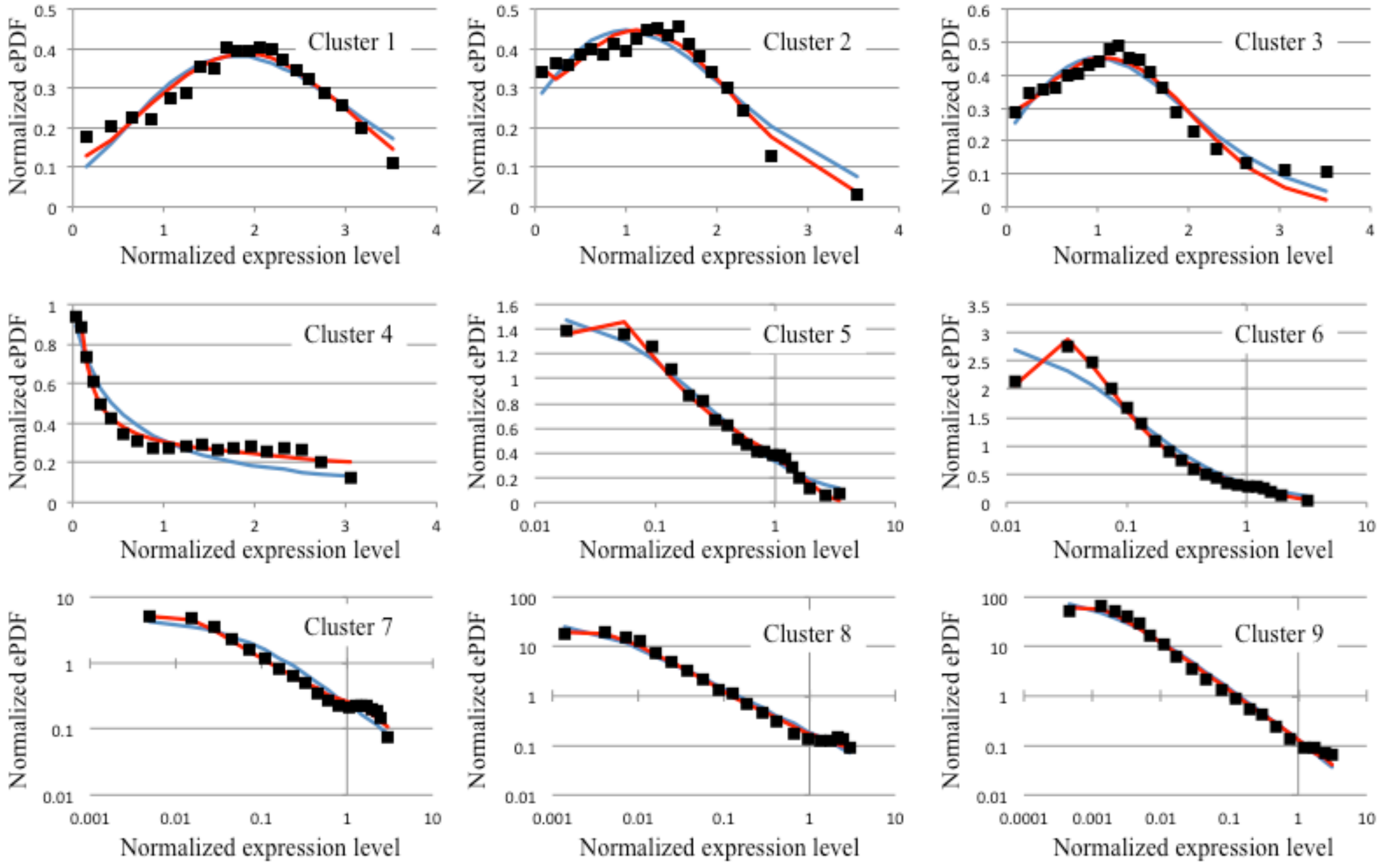
Cluster 1
Cluster 2
Cluster 3
Cluster 4
Cluster 5
Cluster 6
Cluster 7
Cluster 8
Cluster 9
Normalized ePDF
Normalized expression level

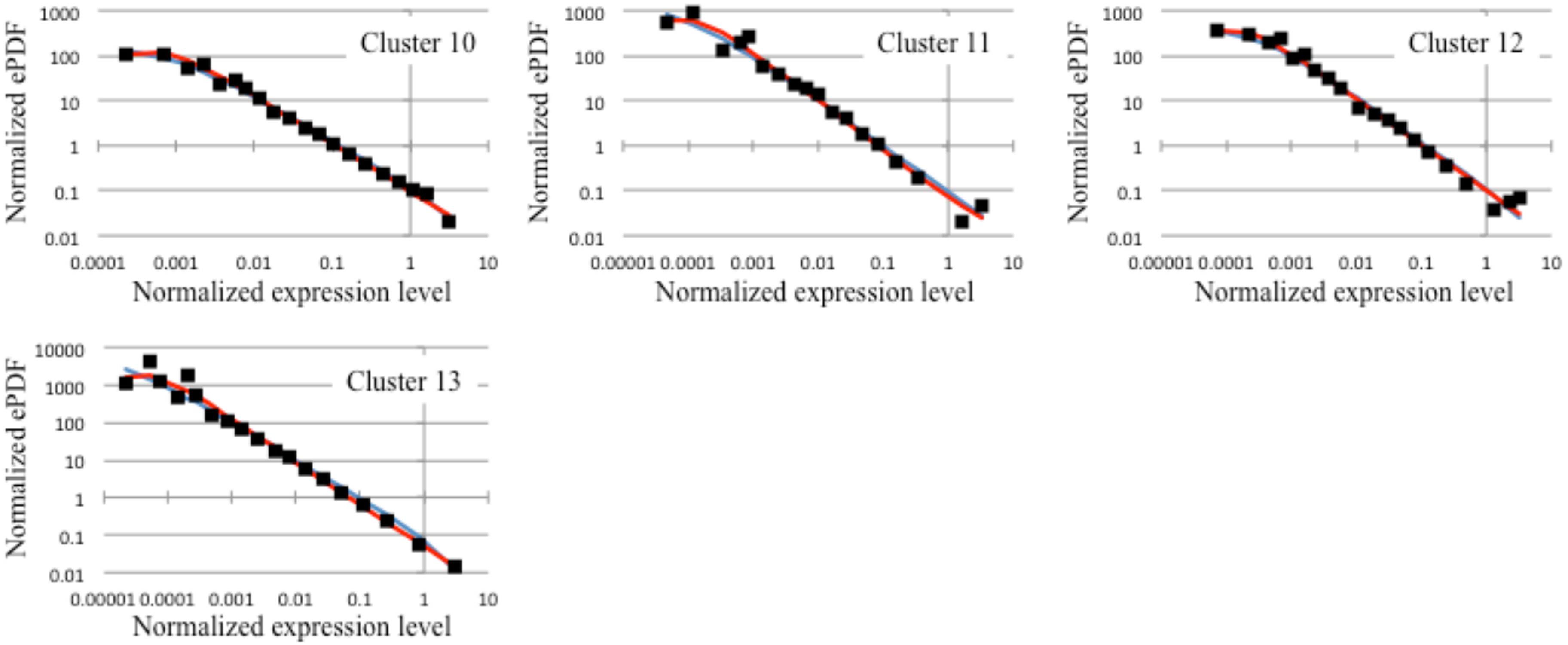

Figure S1. ePDF for indicated clusters in *Arabidopsis* (1 h, 7 days old). Red and blue represent curves fitted with the G-P and NB distribution functions, respectively.

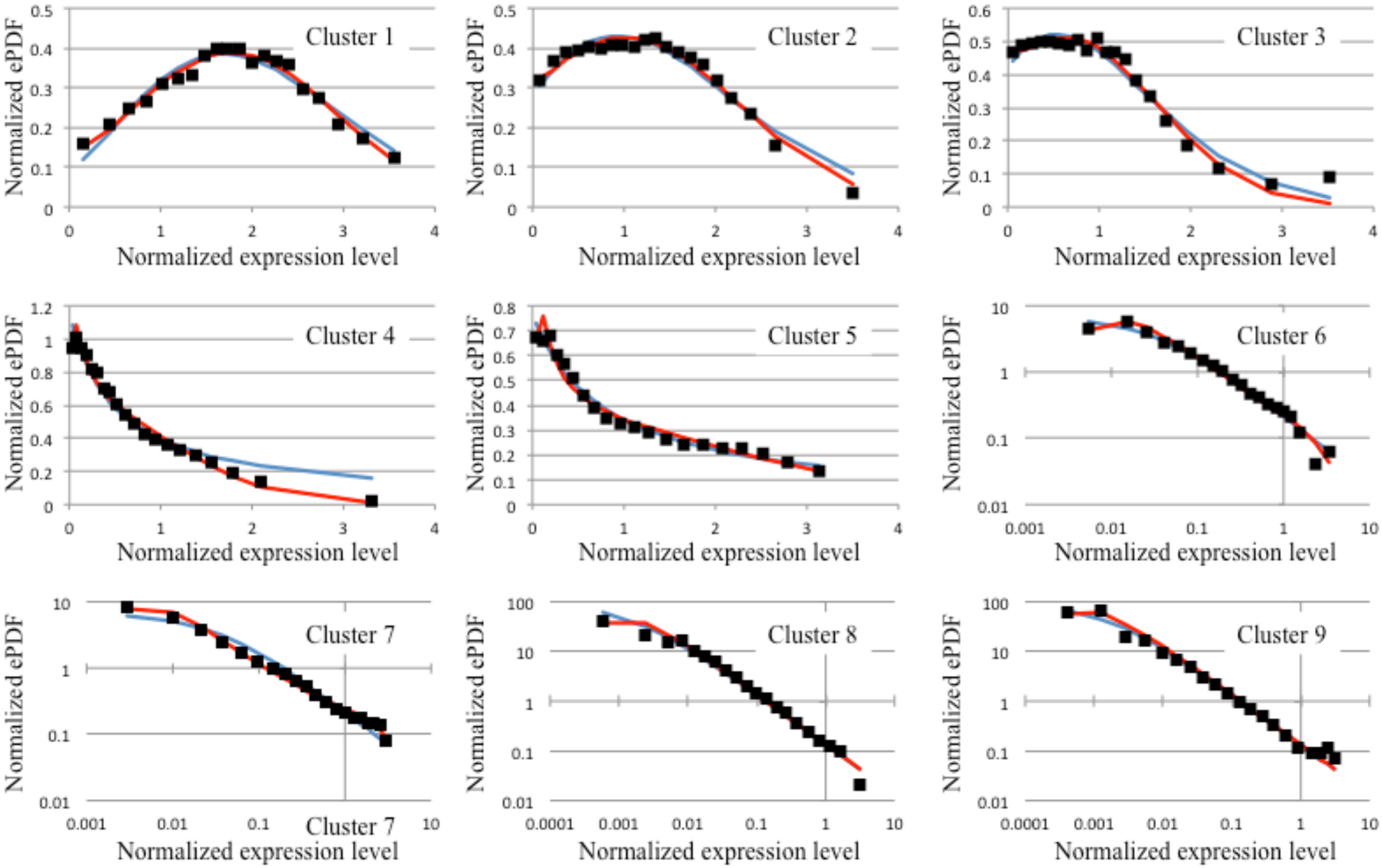
Cluster 1
Normalized ePDF
0 0.1 0.2 0.3 0.4 0.5
0 1 2 3 4
Normalized expression level
Cluster 2
Normalized ePDF
0 0.1 0.2 0.3 0.4 0.5
0 1 2 3 4
Normalized expression level
Cluster 3
Normalized ePDF
0 0.1 0.2 0.3 0.4 0.5 0.6
0 1 2 3 4
Normalized expression level
Cluster 4
Normalized ePDF
0 0.2 0.4 0.6 0.8 1 1.2
0 1 2 3 4
Normalized expression level
Cluster 5
Normalized ePDF
0 0.1 0.2 0.3 0.4 0.5 0.6 0.7 0.8
0 1 2 3 4
Normalized expression level
Cluster 6
Normalized ePDF
0.01 0.1 1 10
0.001 0.01 0.1 1 10
Normalized expression level
Cluster 7
Normalized ePDF
0.01 0.1 1 10
0.001 0.01 0.1 Cluster 7 10
Normalized expression level
Cluster 8
Normalized ePDF
0.01 0.1 1 10 100
0.0001 0.001 0.01 0.1 1 10
Normalized expression level
Cluster 9
Normalized ePDF
0.01 0.1 1 10 100
0.0001 0.001 0.01 0.1 1 10
Normalized expression level

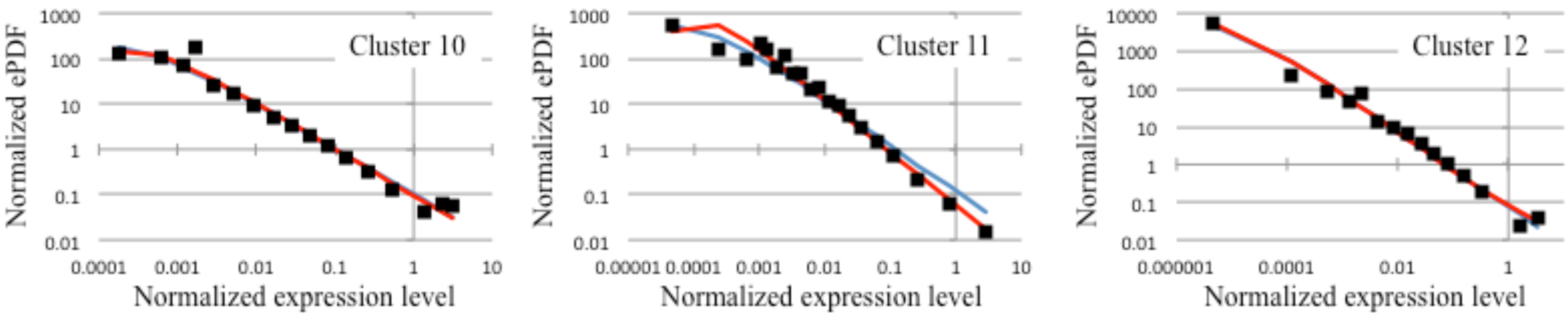

Figure S2. ePDF for indicated clusters in *Arabidopsis* (7 h, 7 days old). Red and blue represent curves fitted with the G-P and NB distribution functions, respectively.

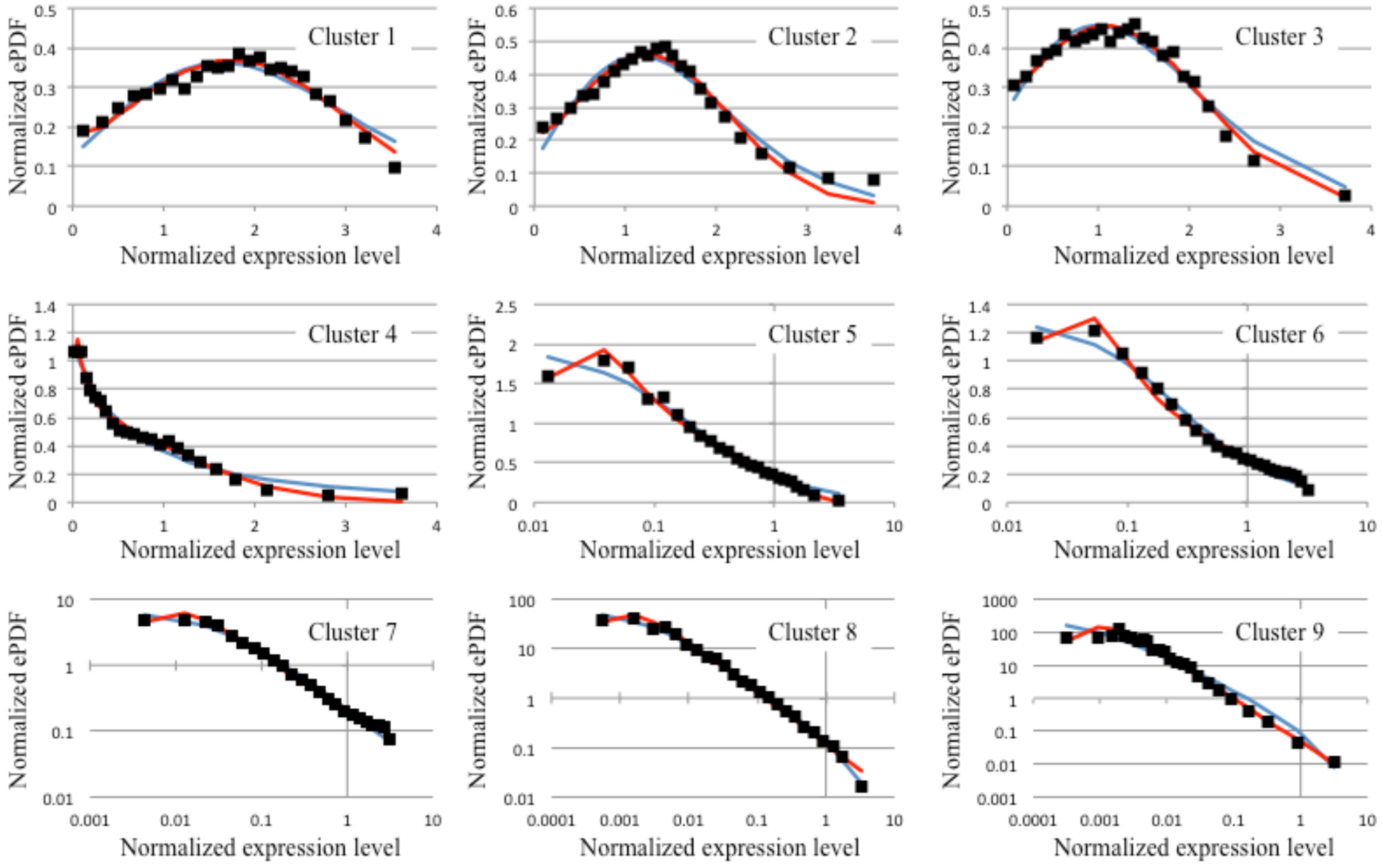
Cluster 1
Cluster 2
Cluster 3
Cluster 4
Cluster 5
Cluster 6
Cluster 7
Cluster 8
Cluster 9
Normalized ePDF
Normalized expression level

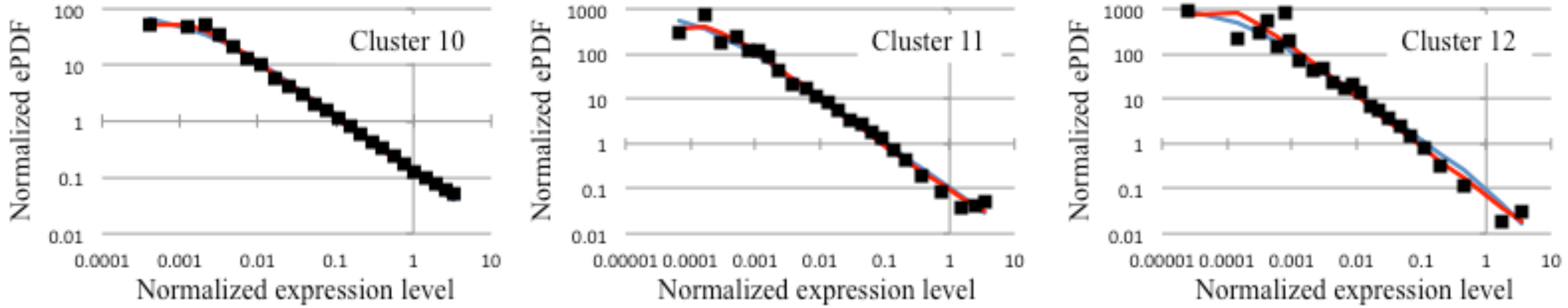

Figure S3. ePDF for indicated clusters in *Arabidopsis* (13 h, 7 days old). Red and blue represent curves fitted with the G-P and NB distribution functions, respectively.

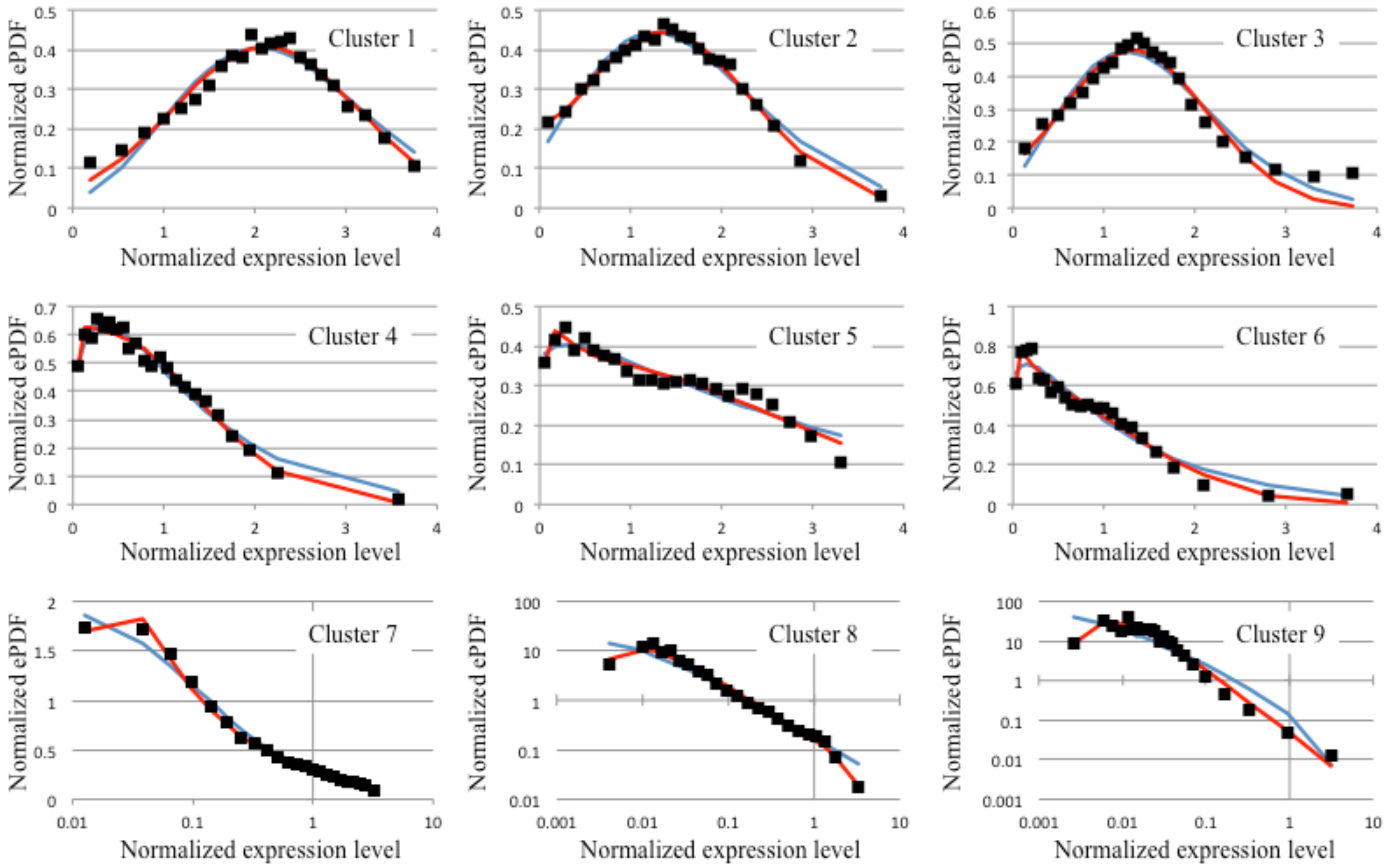

Cluster 1
Cluster 2
Cluster 3
Cluster 4
Cluster 5
Cluster 6
Cluster 7
Cluster 8
Cluster 9
Normalized ePDF
Normalized expression level

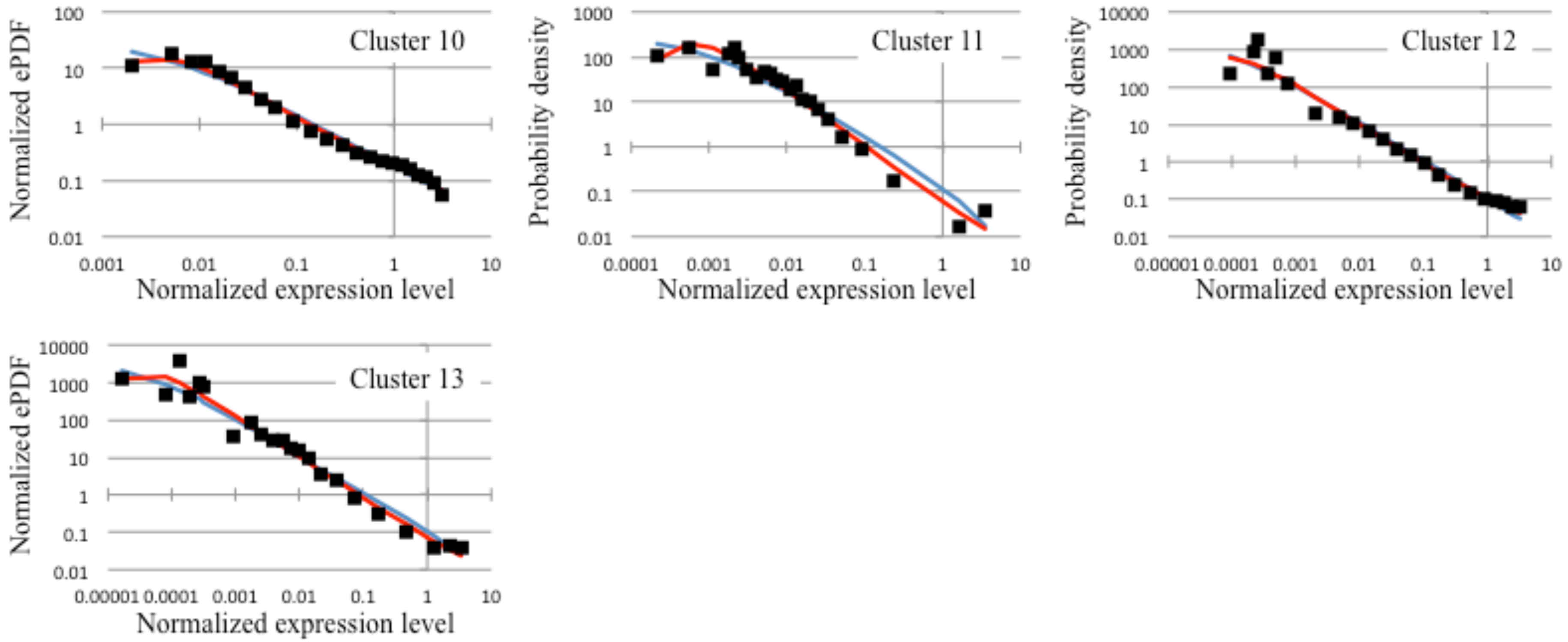

Figure S4. ePDF for indicated clusters in *Arabidopsis* (19 h, 7 days old). Red and blue represent curves fitted with the G-P and NB distribution functions, respectively.

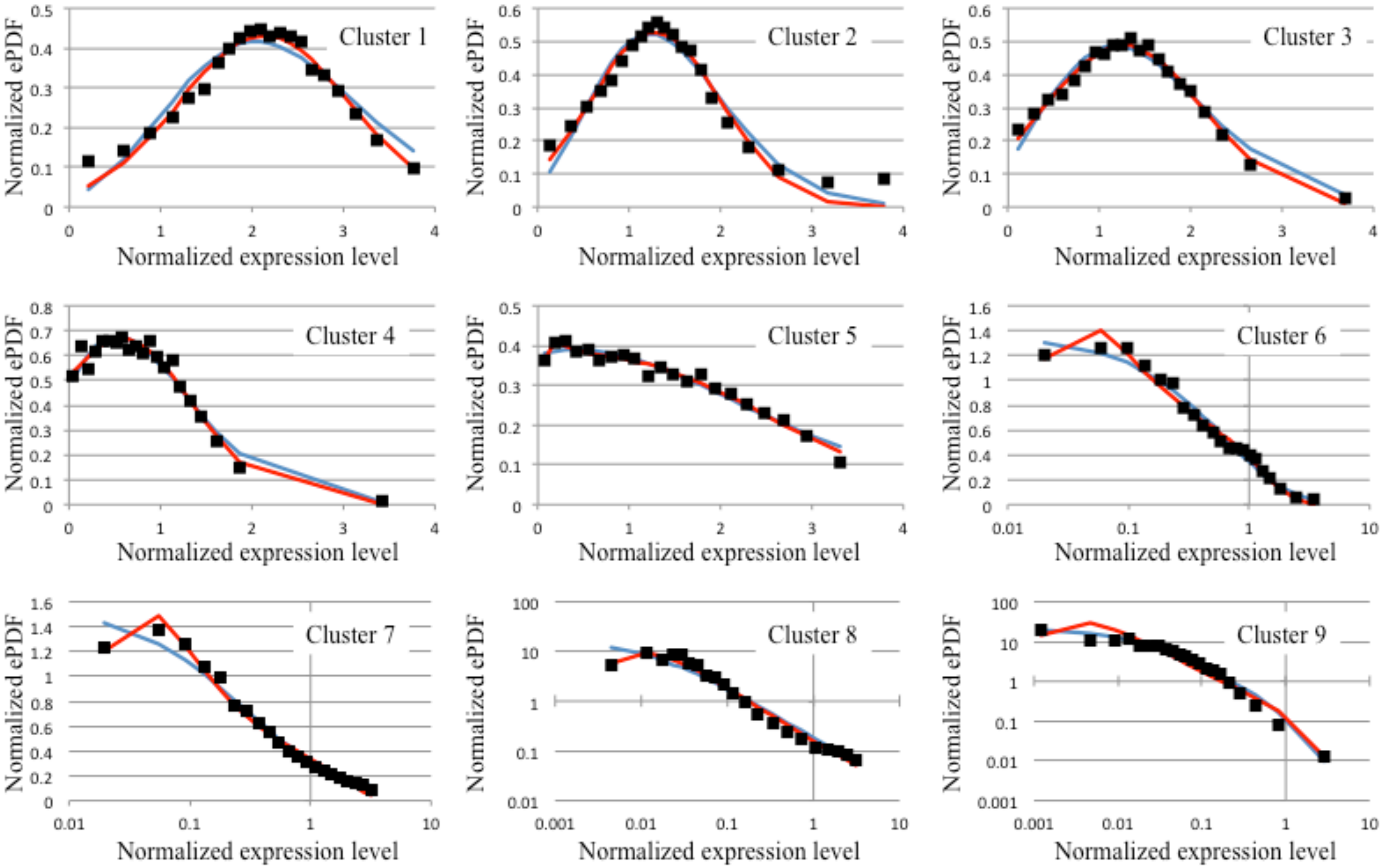
Cluster 1
Cluster 2
Cluster 3
Cluster 4
Cluster 5
Cluster 6
Cluster 7
Cluster 8
Cluster 9
Normalized ePDF
Normalized expression level

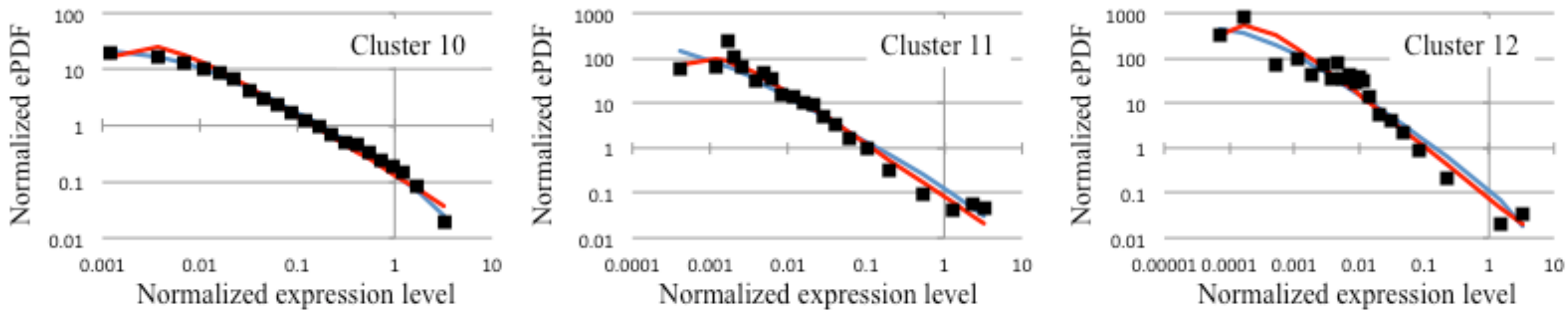

Figure S5. ePDF for indicated clusters in *Arabidopsis* (1 h, 22 days old). Red and blue represent curves fitted with the G-P and NB distribution functions, respectively.

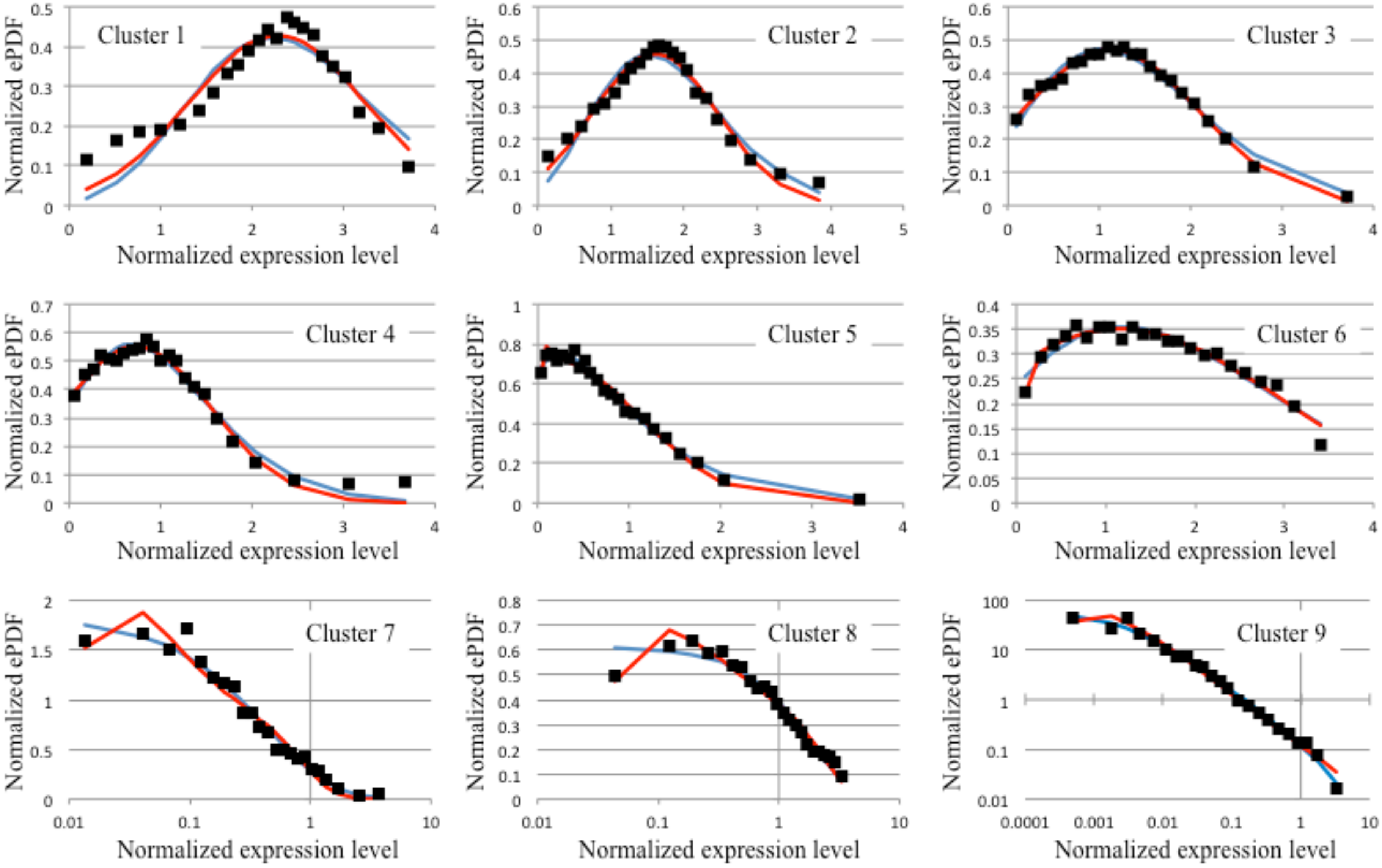
Cluster 1
Cluster 2
Cluster 3
Cluster 4
Cluster 5
Cluster 6
Cluster 7
Cluster 8
Cluster 9
Normalized ePDF
Normalized expression level

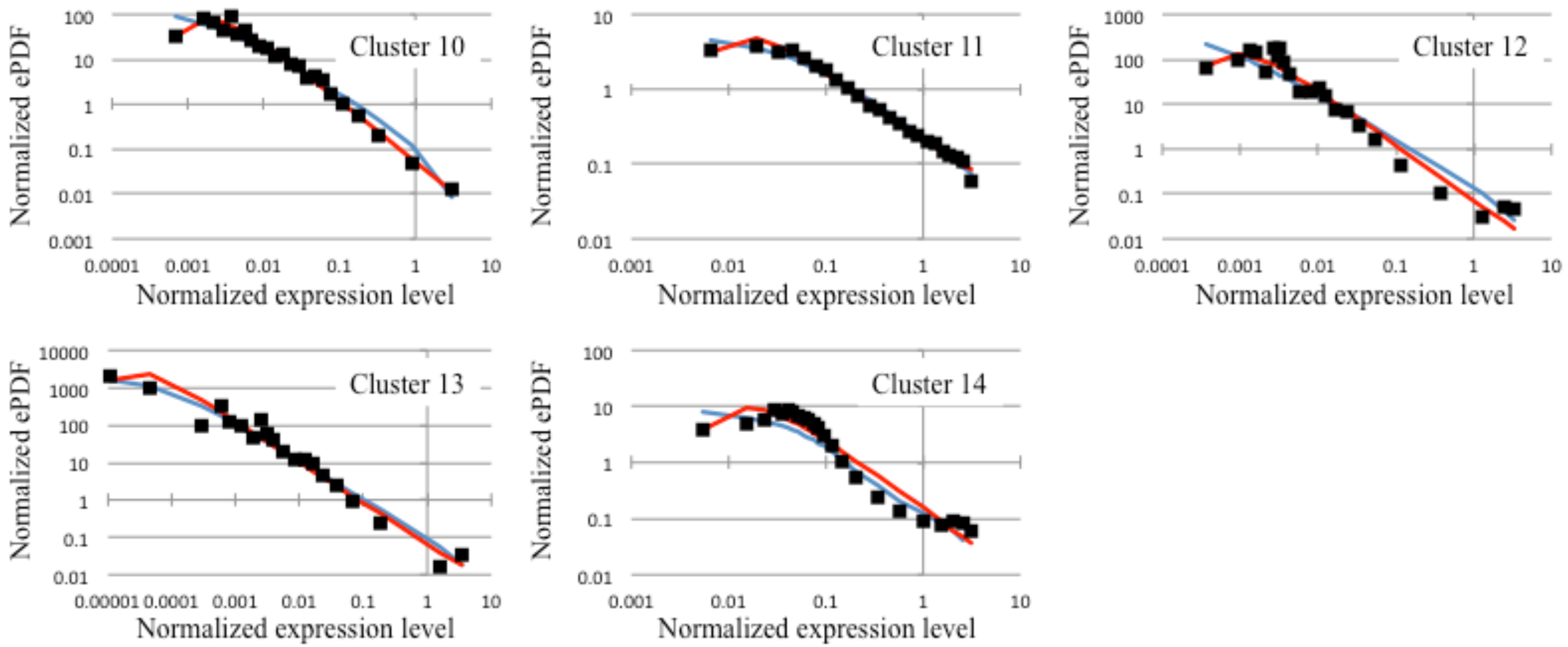

Figure S6. ePDF indicated clusters in *Arabidopsis* (7 h, 22 days old). Red and blue represent curves fitted with the G-P and NB distribution functions, respectively.

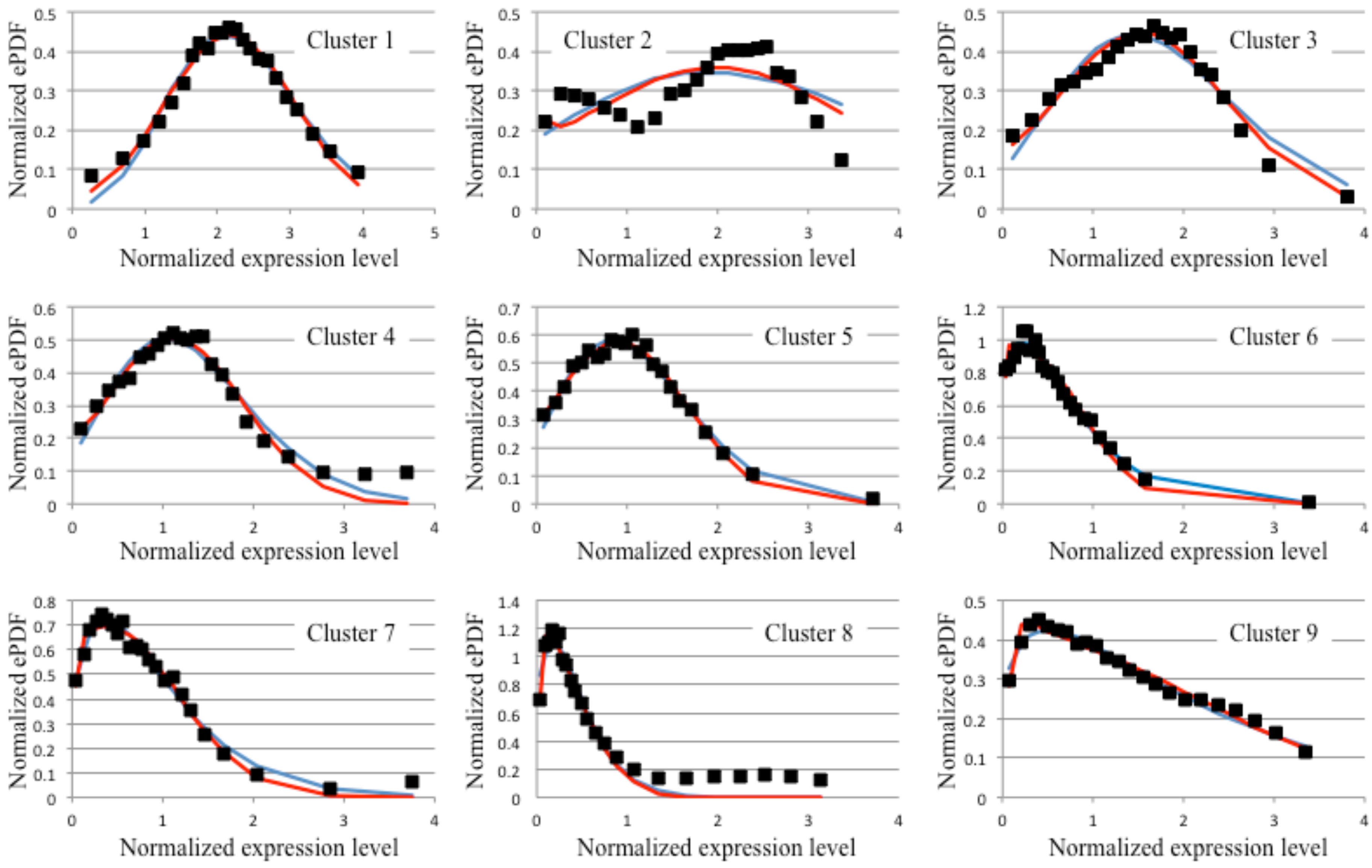
Cluster 1
Cluster 2
Cluster 3
Cluster 4
Cluster 5
Cluster 6
Cluster 7
Cluster 8
Cluster 9
Normalized ePDF
Normalized expression level

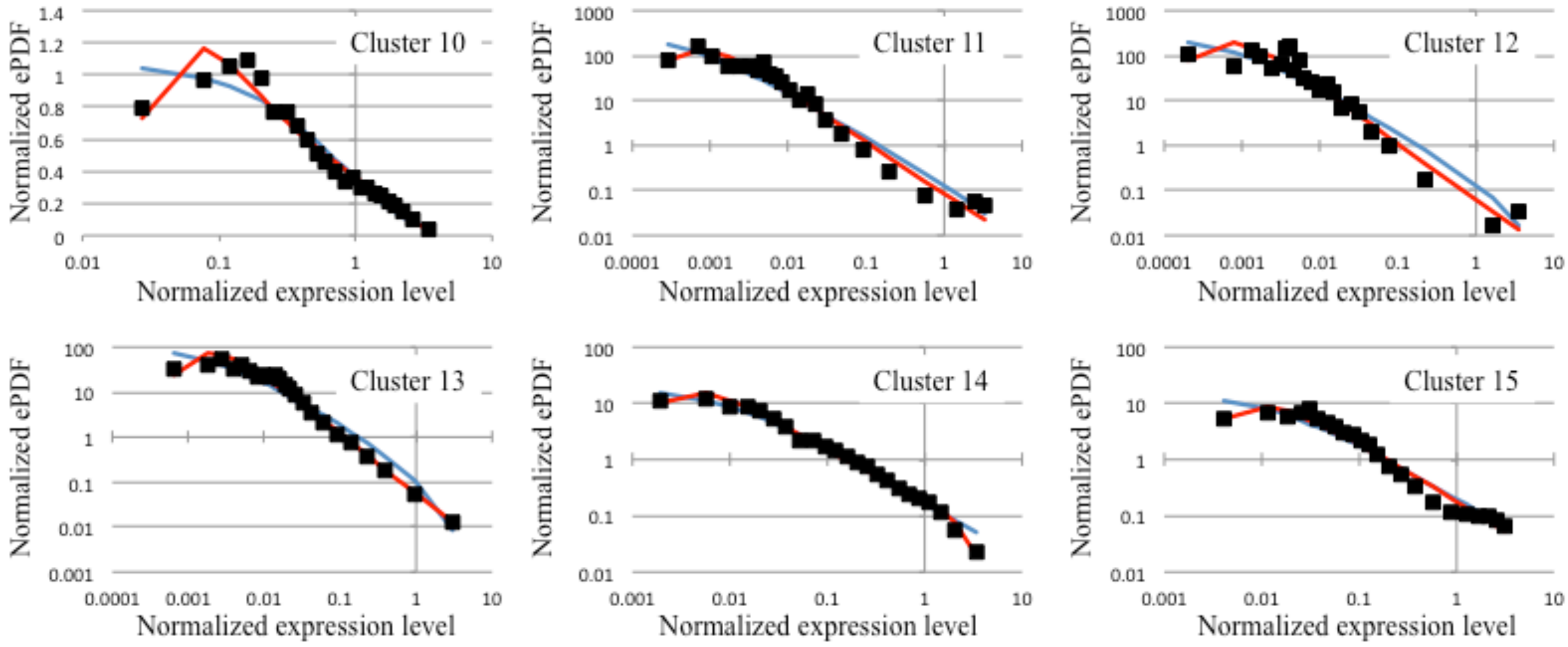

Figure S7. ePDF for indicated clusters in *Arabidopsis* (13 h, 22 days old). Red and blue represent curves fitted with the G-P and NB distribution functions, respectively.

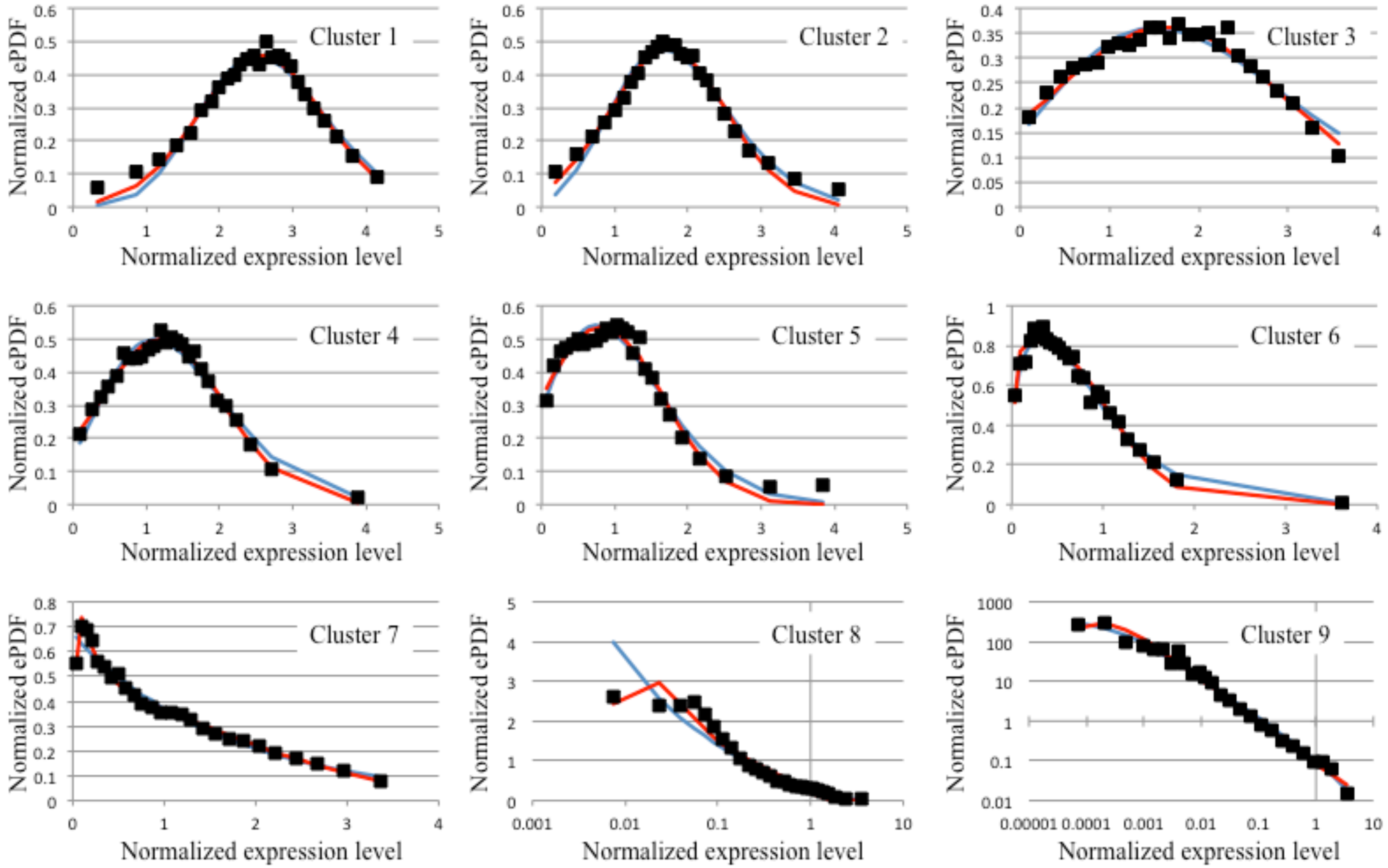

Cluster 1
Cluster 2
Cluster 3
Cluster 4
Cluster 5
Cluster 6
Cluster 7
Cluster 8
Cluster 9
Normalized ePDF
Normalized expression level

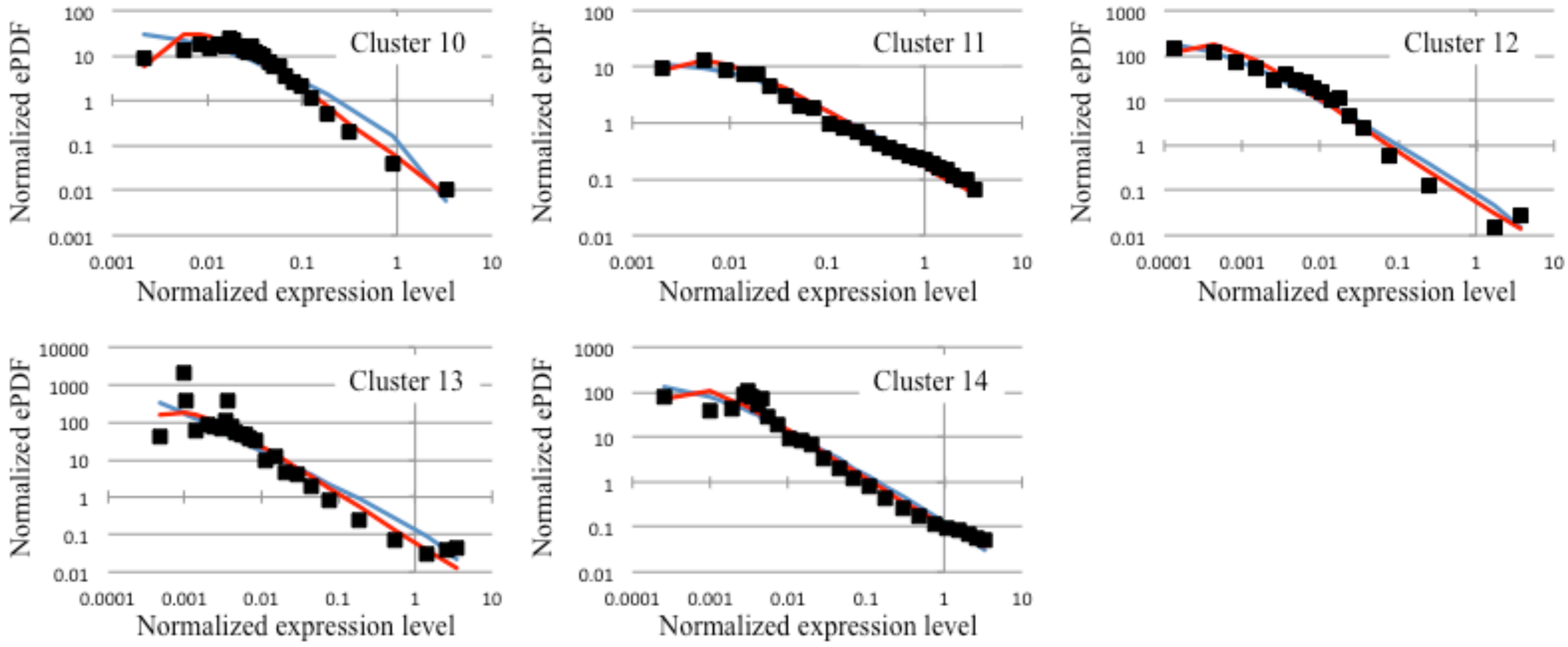

Figure S8. ePDF for indicated clusters in *Arabidopsis* (19 h, 22 days old). Red and blue represent curves fitted with the G-P and NB distribution functions, respectively.

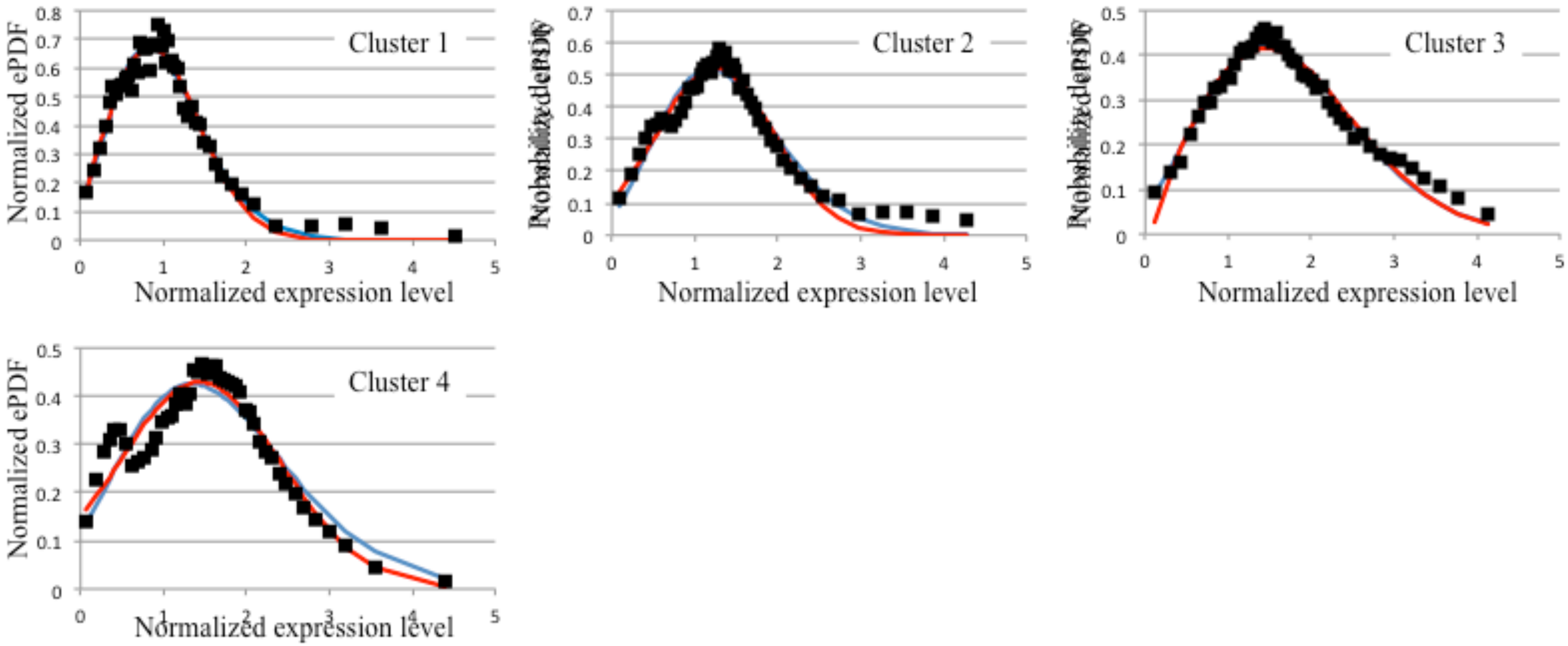

Figure S9. ePDF for indicated clusters in *S. cerevisiae*. RNA-seq data were derived from 48-replicate experiments. Red and blue represent curves fitted with the G-P and NB distribution functions, respectively.

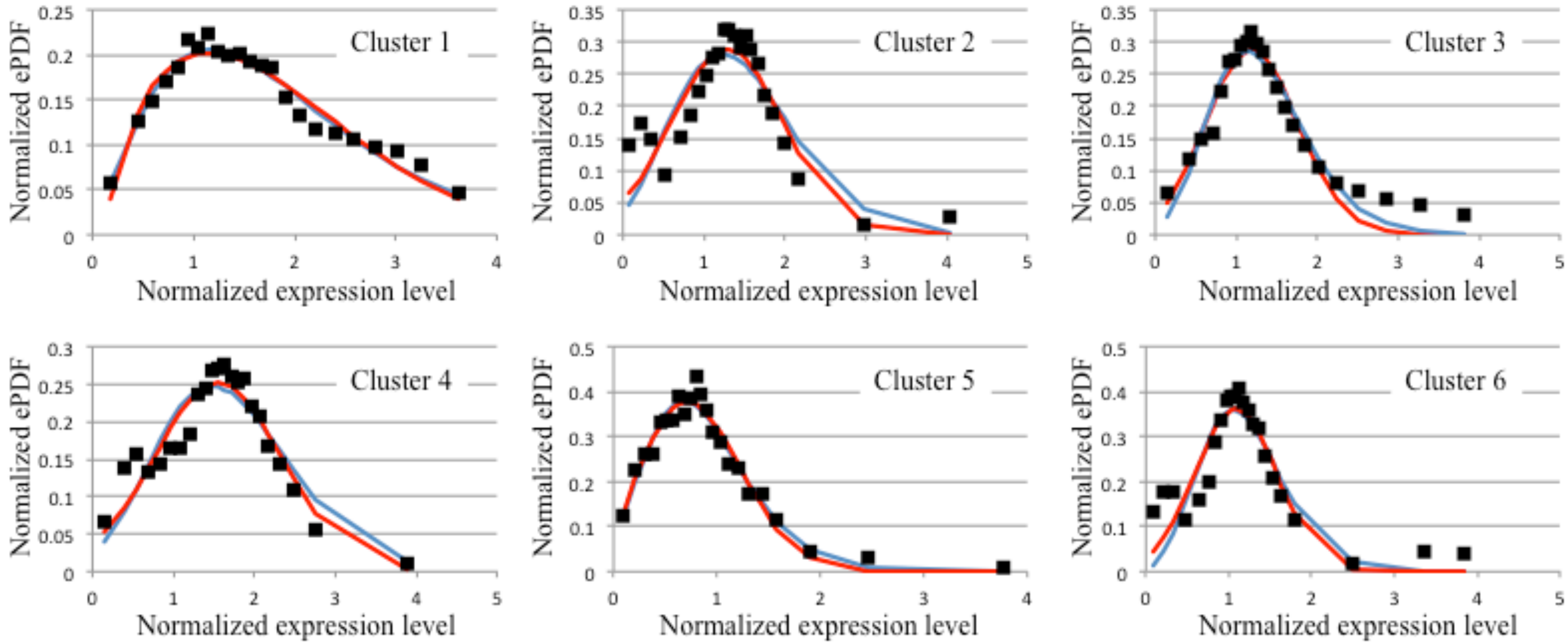

Figure S10. ePDF for indicated clusters in *S. cerevisiae*. RNA-seq data (# of data = 24) were randomly selected from 48-replicate experiments. Red and blue represent curves fitted with the G-P and NB distribution functions, respectively.
